# Metalloprotein-Based Nanomedicines: Design Strategies, Functional Mechanisms, and Biomedical Applications

**DOI:** 10.3390/ijms27021076

**Published:** 2026-01-21

**Authors:** Tingting Ma, Zhongwei Mao, Bin Xue, Yi Cao, Wei Sun

**Affiliations:** 1 Collaborative Innovation Center of Advanced Microstructures, National Laboratory of Solid State Microstructure, Department of Physics, Nanjing University, Nanjing 210093, China; 602022220035@smail.nju.edu.cn (T.M.); xuebinnju@nju.edu.cn (B.X.); 2 Jinan Microecological Biomedicine Shandong Laboratory, Jinan 250021, China; 502023220135@smail.nju.edu.cn

**Keywords:** metal nanomedicine, metalloproteins, protein carriers, multimodal therapy, targeted delivery, theranostics

## Abstract

Metalloprotein-based nanomedicines integrate the multifunctionality of metal centers with the engineerability of proteins to construct advanced nanoplatforms for targeted delivery, diagnostic imaging, and multimodal therapy. In these nanomedicines, metal ions or clusters act as functional cores, enabling imaging contrast enhancement, catalytic reactions, and modulation of pathological microenvironments, while protein frameworks provide structural stability, intrinsic biocompatibility, and programmable bio-interfaces. This review summarizes the design principles of three major metalloprotein-based nanomedicines, including native metalloproteins, engineered metalloproteins, and metal–protein hybrid nanostructures, with a focus on ferritin, transferrin, and heme/cytochrome proteins in the contexts of cancer therapy, imaging diagnostics, antimicrobial, and anti-resistance applications. Through discussion of representative metal- and metalloprotein-based nanomedicine candidates, this review highlights the current challenges and outlines opportunities brought by emerging technologies such as artificial intelligence-guided protein design. Collectively, these advances underscore metal- and metalloprotein-based nanomedicines as multifunctional, tunable, and clinically promising platforms that are poised to become an important pillar of future nanomedicine.

## 1. Introduction

Metals and their complexes are widely present in living systems and play important roles in many biological processes, from metabolic catalysis and oxygen transport to the regulation of immune responses and signal transduction [1,2,3]. In nanomedicine, metal ions and metal clusters are not merely passive cargo encapsulated within carriers but instead serve as the functional core that governs material behavior [4]. Owing to their distinctive electronic structures and coordination chemistry, transition metals and selected lanthanides play irreplaceable roles in redox catalysis, regulation of reactive oxygen species (ROS), energy conversion, and physical imaging [5,6,7]. Therapeutic and diagnostic modalities such as Fenton and Fenton-like catalytic therapy, photothermal and photoacoustic treatments based on localized surface plasmon resonance or non-radiative relaxation, as well as magnetic resonance and computed tomography (CT) imaging that rely on high atomic number X-ray attenuation, all fundamentally depend on the quantum states, valence transitions and coordination environments of metal centers [8,9,10]. 

Compared with purely organic small molecules or polymeric carriers, metals can concurrently serve as imaging signal sources, local energy deposition centers, and catalytic hotspots within a single nanoscale construct, thereby providing a unique physicochemical foundation for the development of integrated theranostic platforms.

Within the broader field of metal-based nanomedicine, purely inorganic or polymer–metal nanoplatforms have been intensively studied. Representative inorganic nanomaterials, including gold [11,12], silver [13,14], ferrites [15,16,17,18], and other metal oxides, have shown outstanding performance in photothermal and photodynamic therapy as well as multimodal imaging [19,20]. Porous materials typified by metal–organic frameworks (MOFs) further offer high loading capacity and well-defined channels for stimuli-responsive drug release [21,22]. However, these systems share several intrinsic limitations, including prolonged in vivo retention, limited biodegradability, and restricted programmability of their biointerfaces [23,24,25]. In contrast, metal- and metalloprotein-based nanomedicines, defined here as nanoscale systems in which metal centers are functionally integrated with native or engineered protein scaffolds to actively regulate metal coordination, reactivity, or biological interactions—rather than being passively coated by proteins or incidentally acquiring a protein corona—are expected to retain the imaging and therapeutic functions of metals while exploiting the advantages of proteins in spatial confinement, microenvironment modulation, and biointerface engineering. This approach promises a more favorable balance between therapeutic efficacy and biosafety [26].

Proteins possess highly ordered and programmable three-dimensional architectures, inherent biocompatibility, and rich opportunities for fine-tuning by genetic engineering and chemical modification, making them particularly well suited for dynamic and stimuli-responsive regulation of metal coordination and activity in complex biological environments [27]. In this context, recent studies have increasingly focused on redox- and glutathione (GSH)-responsive metal–protein nanocarriers, which exploit the distinct redox gradients between extracellular environments and intracellular compartments, particularly in tumors and inflamed tissues. By leveraging elevated intracellular GSH levels or aberrant redox states, such systems enable conditional activation of metal-mediated catalysis, controlled metal release, or on-demand imaging signal amplification, thereby mitigating off-target toxicity associated with constitutively active metal species. These advances underscore the growing recognition that precise regulation of metal speciation and reactivity—rather than metal loading alone—is central to the design of safe and effective metal-based nanomedicines [28,29,30]. Such scaffolds can provide stable confined spaces and tunable coordination environments for metal ions or clusters [31,32] and, through receptor-targeting ligands, cell-penetrating peptides and diverse stimuli-responsive units, markedly improve the pharmacokinetics and tissue distribution of nanomedicines [33,34,35]. Building on these features, a variety of representative metal–protein systems have emerged. These include native metalloprotein platforms based on ferritin [36], transferrin [37], hemoglobin and cytochromes [38,39]; engineered metalloproteins constructed via site-directed mutagenesis, metal-ion-induced self-assembly and coordination-driven assembly [40]; and metal–protein hybrid nanostructures prepared by protein coating, protein-templated mineralization or the formation of metal–protein frameworks (MPFs) [41,42]. These systems have demonstrated considerable potential in cancer therapy [43], imaging diagnostics [44], and antimicrobial and anti-resistance treatments [45]. Nevertheless, their complex in vivo behavior, the detailed mechanisms at metal–protein interfaces, and critical issues for clinical translation require systematic clarification.

**Figure 1:**
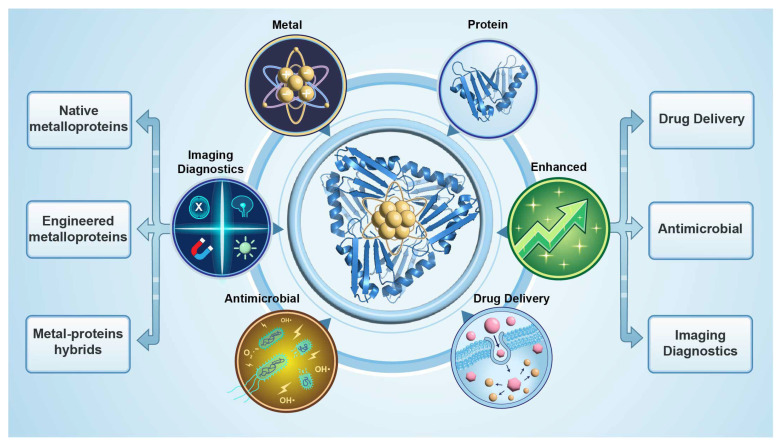
Schematic overview of metal–protein nanomedicines, including material design strategies, structural classifications, and representative biomedical applications.

In this review, we first summarize the major types of metal- and metalloprotein-based nanomedicines and their construction strategies and then highlight recent advances in biomedical applications, with an emphasis on tumor treatment, imaging diagnosis, and anti-infective therapy (Figure 1). We further discuss common challenges related to structural characterization, interface mechanisms, process scale-up, and safety evaluation, aiming to distill general design principles for future material development. Finally, we outline emerging opportunities brought by artificial-intelligence-assisted protein design for the fine regulation of metal–protein interfaces and prediction of in vivo behavior, and consider how these advances may collectively accelerate the clinical translation of metal- and metalloprotein-based nanomedicines.

## 2. Construction Mechanisms of Metalloprotein-Based Nanomedicines

Metal–protein interactions underlie many essential life processes, thus providing metal–protein combinations a natural advantage in the design of nanomedicines. According to how metals associate with proteins, the structural complexity and the degree of functional integration reflect the increasing levels of design sophistication and multifunctionality. The current metalloprotein-based nanomedicines are mainly constructed based on the following mechanisms: (i) native metalloproteins that are directly used in their physiological forms; (ii) engineered metalloproteins obtained by protein engineering and chemical modification; and (iii) metal–protein hybrid nanostructures constructed from metals and proteins as separate building blocks. These three classes represent a continuum ranging from naturally evolved structures, through rationally redesigned proteins, to fully hybrid functional materials. In this section, we summarize their key structural features, metal-binding mechanisms, and typical construction strategies.

### 2.1. Native Metalloproteins

Native metalloproteins are ubiquitously present in living organisms. Their metal-binding sites have been refined by biological evolution and typically feature specific coordination environments, stable tertiary or quaternary structures, and good physiological compatibility. As such, they represent one commonly foundational module for constructing metal–protein nanomedicines. Typical examples include ferritin, transferrin, and other cofactor-containing proteins such as hemoglobin and cytochromes. Leveraging the intrinsic structural and functional advantages of these proteins, researchers have developed multifunctional designs for drug loading, metal ion delivery, catalytic therapy, and imaging enhancement. From a design perspective, native metalloproteins offer several generalizable principles for constructing metal–protein nanomedicines. These include (i) interface-defined metal coordination environments that constrain metal speciation and reactivity, (ii) a balance between structural stability and functional activation that is often condition-dependent, and (iii) the use of endogenous biological pathways, such as metal transport or receptor-mediated uptake, to couple therapeutic function with physiological compatibility.

**Figure 2:**
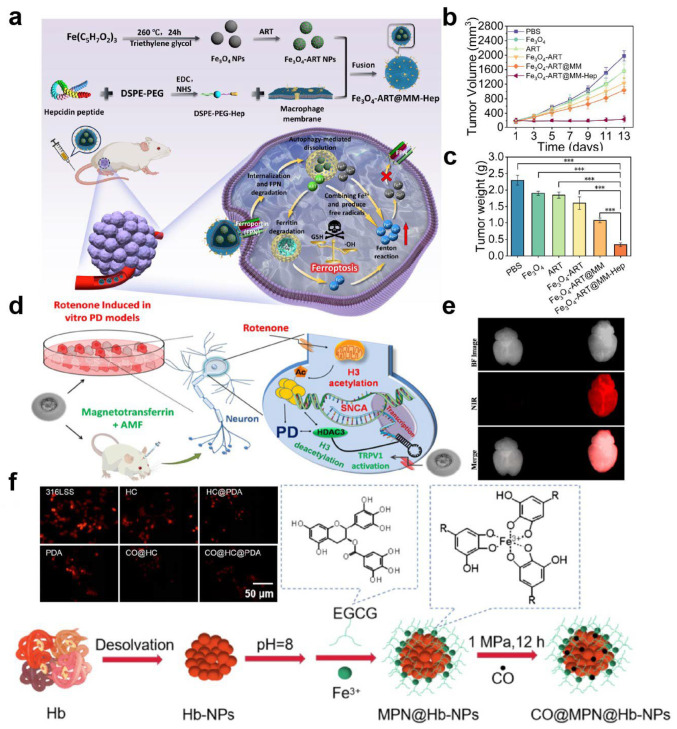
Representative native metalloprotein-based nanoplatforms for metal delivery and therapeutic modulation. (**a**) Schematic illustration of the preparation of Fe_3_O_4_-ART@MM-Hep nanoparticles and the ferroportin-hijacking strategy for enhanced ferrotherapy via intracellular Fe^2+^ elevation. (**b**) Tumor volume changes during treatment in different groups. (**c**) Average tumor weights at the end of treatment. Reproduced with permission. *** *p* < 0.001 [49]. Copyright 2025, American Chemical Society. (**d**) Schematic illustration of reconstituted magnetotransferrin formed by nucleation of Fe_2_O_3_ nanocrystals within the iron-binding clefts of apotransferrin. (**e**) Ex vivo brain reflectance imaging showing enhanced accumulation of ICG-labeled magnetotransferrin. Reproduced with permission [58]. Copyright 2023, American Chemical Society. (**f**) Schematic illustration of the preparation of hemoglobin/epigallocatechin-3-gallate (EGCG) core–shell nanoparticles and CO-loaded CO@Hb/EGCG coatings for inflammation modulation. Reproduced with permission [65]. Copyright 2024, Regenerative Biomaterials.

Ferritin has been extensively explored as a nanomedicine platform owing to its hollow cage-like architecture and excellent metal encapsulation capacity under controlled experimental conditions [46]. Human ferritin consists of 24 self-assembled subunits that form a protein shell with an outer diameter of ~12 nm and an internal cavity of ~8 nm. Through controlled mineralization, this cavity can be loaded with iron and a variety of transition metal nanoclusters, giving rise to metal-core–protein-shell nanostructures [36,46]. Ferritin is highly water-soluble and is generally regarded as biocompatible based on extensive in vitro studies and selected in vivo models. Moreover, a wide range of metal species and therapeutic agents have been successfully encapsulated within ferritin nanocages [47,48]. Zhao et al. constructed a ferritin/ferroportin-hijacking nanoplatform (Fe_3_O_4_-ART@MM-Hep), featuring an Fe_3_O_4_–artemisinin core cloaked with hepcidin-modified macrophage membrane (Figure 2a) [49]. After tumor accumulation, hepcidin binds ferroportin to trigger nanoparticle/FPN internalization and degradation, leading to Fe_3_O_4_ disassembly, artemisinin release, ferritin degradation, and thereby massive Fe^2+^ release. By simultaneously enhancing exogenous iron input, mobilizing endogenous iron stores, and blocking iron efflux, this system amplifies ferroptosis in the reported tumor models, illustrating that ferritin- and transferrin-based systems can outperform non-targeted or purely synthetic nanocarriers primarily under conditions where receptor availability, vascular access, and intracellular processing remain favorable (Figure 2b,c). Collectively, ferritin-based systems illustrate a key design principle for native metalloproteins: robust protein architectures can serve as confinement-defined nanoreactors that stabilize metal species under controlled conditions while enabling functional activation through regulated disassembly, degradation, or metal release in specific biological contexts.

Transferrin is a glycoprotein mainly synthesized by the liver and secreted into the bloodstream, where it is responsible for iron transport. It binds with high affinity to transferrin receptors, which are highly expressed in many types of cancer [50,51,52]. Consequently, transferrin can be used both as a carrier and as a targeting ligand. In certain tumor models, this dual-function strategy has been shown to enhance receptor-mediated uptake [53,54], which may translate into improved tissue distribution of nanomedicines in vivo, depending on receptor expression levels and disease context [55]. Beyond serving as a stabilizing carrier and targeting ligand, the inherent metal-binding capacity of transferrin has been exploited to develop iron-metabolism-responsive therapeutic systems, particularly for ferroptosis regulation and tumor microenvironment remodeling [56]. For example, Gao et al. demonstrated that serum transferrin is indispensable for ferroptosis by delivering iron into cells via transferrin receptor-mediated endocytosis, thereby providing the iron prerequisite for glutaminolysis-driven, cystine starvation-induced ferroptotic cell death [57]. In addition, Sardoiwala et al. constructed a reconstituted magnetotransferrin by nucleating Fe_2_O_3_ nanocrystals within the iron-binding clefts of apotransferrin, enabling transferrin receptor-mediated brain targeting and alternating-magnetic-field-induced mild magnetothermal activation of the TRPV1–HDAC3 axis to reduce α-synuclein aggregation and ameliorate Parkinson’s neurobehavioral deficits (Figure 2d,e) [58]. In addition, sequestration of labile iron by transferrin has been used to inhibit the growth of various pathogens, including Gram-negative bacteria such as Pseudomonas aeruginosa [37,59,60]. Despite their frequent designation as “ideal” targeting carriers, ferritin- and transferrin-based systems exhibit context-dependent performance in vivo. Receptor-mediated uptake can be significantly compromised by receptor saturation, competition with abundant endogenous ligands, and heterogeneous receptor expression across tumor types and disease stages. In addition, altered receptor recycling and impaired vascular accessibility in advanced tumors may further limit the effectiveness of these targeting mechanisms [61]. As a result, the in vivo advantage of ferritin- or transferrin-mediated targeting is not universal and may diminish under conditions of high endogenous protein levels or poor tumor perfusion [62]. These observations highlight a broader design principle for receptor-targeted metalloprotein nanomedicines: the effectiveness of biologically inspired targeting strategies is inherently constrained by endogenous ligand competition, receptor dynamics, and disease-stage-dependent microenvironmental accessibility.

Furthermore, heme-containing proteins, such as hemoglobin and cytochromes, are increasingly being incorporated into the design of metal–protein nanomedicines. Hemoglobin can participate in photodynamic therapy by supplying oxygen and exploiting the redox activity of the heme center to promote ROS generation [38,63]. In addition to photodynamic therapy (PDT), hemoglobin-based nanoplatforms have also been engineered as oxygen carriers to overcome tumor hypoxia. For example, Gandhi et al. developed hemoglobin-loaded polycaprolactone nanoparticles (PCL–Hb NPs) that preserve hemoglobin’s oxygen-binding capacity, exhibit acceptable biocompatibility within the tested in vitro and in vivo models, and are efficiently internalized by tumor and stromal cells under both normoxic and hypoxic conditions [64]. By delivering oxygen into hypoxic tumor cells, PCL–Hb NPs downregulate hypoxia-responsive genes and effectively reverse paclitaxel resistance, thereby enhancing chemotherapeutic efficacy in 2D hypoxic cultures and 3D tumor spheroids. In addition, hemoglobin nanocarriers can be used to load and release gaseous transmitters via metal-centered coordination. Wu et al. engineered a hemoglobin/epigallocatechin-3-gallate (EGCG) core–shell nanoparticle coating on stainless steel that stores CO through hemoglobin–CO coordination to modulate implantation-induced inflammation (Figure 2f) [65]. This coating provides sustained CO release and has been shown to lower intracellular ROS levels and attenuate oxidative stress-induced apoptosis in endothelial cells under the tested conditions. In vivo, the hemoglobin/EGCG-based CO-releasing coating suppresses macrophage-mediated inflammatory responses and favorably regulates macrophage polarization at the implant interface. Cytochromes, with their unique electron-transfer and redox properties, can be leveraged to modulate the tumor microenvironment or intervene in metabolic pathways [39]. Heme-containing proteins exemplify a broader stability–activity trade-off in metalloprotein-based nanomedicine design, where highly reactive metal cofactors must be carefully spatially confined or temporally activated to balance therapeutic efficacy with oxidative safety. Although these proteins are not yet as widely used as ferritin and transferrin, their distinctive metal cofactor chemistry enriches the toolbox for building metal–protein nanoplatforms.

### 2.2. Metal-Engineered Proteins

In contrast to the direct use of native metalloproteins, metal-engineered proteins place greater emphasis on redesigning metal-binding sites and their local microenvironments on pre-existing protein scaffolds via site-directed mutagenesis, chemical modification, or the introduction of artificial ligands [66]. By adding or substituting coordinating residues such as His, Cys, Asp, and Glu at key positions, or by attaching small-molecule ligands to side chains, one can selectively tune the affinity, coordination number, and geometry of metal ions [67,68], thereby modulating their redox potentials, substrate specificity, and catalytic efficiency. From a design standpoint, metal-engineered proteins represent a shift from evolutionarily constrained metal utilization to rationally tunable systems, where expanded functional space is gained at the cost of increased sensitivity to folding stability, metal exchange, and immune recognition in vivo. Aires et al. fused an Hsp90-binding tetratricopeptide repeat module with a cysteine-rich metal-nanocluster-stabilizing module to create a chimeric protein that nucleates highly fluorescent Au nanoclusters while preserving high-affinity target recognition. The resulting protein–AuNC hybrid acted as a theranostic biologic in an angiotensin II-induced myocardial fibrosis model. It simultaneously reduced cardiac fibrosis and enabled high-sensitivity tracking of drug biodistribution by fluorescence and synchrotron X-ray fluorescence imaging [69].

Similar strategies have been applied to proteins or minimalist peptide scaffolds lacking natural metal cofactors, enabling the construction of artificial metal centers and converting otherwise ordinary proteins into artificial metalloenzymes with defined catalytic, imaging, or signal-amplification functions [70,71,72]. Aires et al. engineered consensus tetratricopeptide repeat (CTPR) proteins with defined acidic, histidine, or cysteine metal-coordination sites to template iron oxide nanoparticles, thereby controlling core size, Fe(III)/Fe(II) ratio, and magnetic relaxivities [73]. By varying both the nature and the number of metal-binding modules, they were able to tune these protein-stabilized iron oxide nanoparticles between T_1_- and T_2_-weighted MRI contrast regimes, highlighting that the design of coordination environments on minimalist protein scaffolds can precisely dictate inorganic core properties and imaging performance. These engineered metalloproteins generally have relatively small molecular weights and well-defined conformations, which facilitates rational optimization based on structure–function relationships, although increased design freedom often comes at the cost of reduced in vivo predictability, including altered folding stability, unexpected immune recognition, and dynamic metal exchange under physiological conditions, due to altered protein stability, unexpected immune recognition, and dynamic metal exchange in physiological environments [74].

Beyond site-specific metal coordination, metal ions are frequently employed at higher structural levels as reversible crosslinkers to induce the self-assembly of engineered proteins [42]. When multiple His residues or short metal-binding peptide motifs are displayed on the protein surface, the addition of related ions under mild conditions can bridge multiple protein units, driving them to assemble from dispersed monomers into size-controlled nanoparticles, cage-like architectures, or physically crosslinked hydrogels [75,76,77]. Liu et al. demonstrated that partially hydrolyzed α-lactalbumin peptides can be directed by various metal ions (Mn^2+^, Co^2+^, Ni^2+^, Zn^2+^, Cd^2+^, and Au^3+^) to self-assemble into hollow nanotubes, which further form supramolecular metallogels with tunable length, stiffness, and gelation kinetics. These results highlight how the identity and coordination strength of metal ions can dictate higher-order protein architectures and their mechanical properties [78]. Owing to the partially reversible nature of metal coordination, these self-assembled systems are often sensitive to mechanical stress, pH, ionic strength, or redox conditions [79] and have been reported to undergo aggregation–disaggregation or sol–gel transitions in response to specific microenvironmental cues, such as pH or ionic strength, under defined conditions, including tumors, inflamed tissues, or intracellular compartments. This behavior has been exploited to design injectable protein hydrogels, environment-responsive carriers, and confined catalytic platforms [40].

### 2.3. Metal–Protein Hybrid Nanostructures

Unlike the molecular-level engineering of individual proteins, metal–protein hybrid nanostructures focus on building intrinsic hybrid nanomedicines. In such systems, proteins and metal nanostructures are treated as two functionally distinct building blocks. The building blocks of metal–protein hybrid nanostructures should be integrated into a single cooperative entity, thus with specific functionalities and great stability through coating, templating, mineralization, or framework assembly. From a design perspective, metal–protein hybrid nanostructures follow an emergent-function principle, in which therapeutic or imaging performance arises from mesoscale spatial integration rather than discrete coordination sites, but this advantage is inherently accompanied by increased structural complexity, batch-to-batch variability, and challenges in in vivo predictability. Depending on the construction strategy, these materials can be broadly categorized into protein-coated metal nanoparticles [80], protein-templated metal nanoclusters [81], and metal–protein framework materials [82].

**Figure 3:**
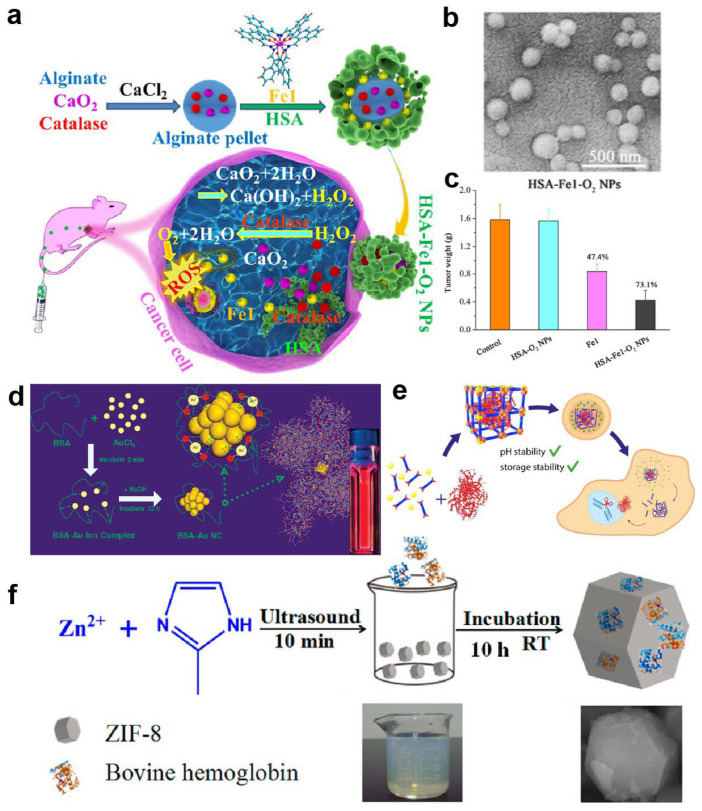
Representative metal–protein hybrid nanostructures. (**a**) Schematic illustration of the protein-coated metal nanoplatform HSA–Fe1–O_2_ NPs. (**b**) Representative morphology characterization of HSA–Fe1–O_2_ NPs. (**c**) Tumor weight in mice treated with HSA–O_2_ NPs, Fe1, or HSA–Fe1–O_2_ NPs [91]. Copyright 2024, American Chemical Society. (**d**) Schematic illustration of protein-templated synthesis of gold nanoclusters (Au NCs) within a BSA matrix. Reproduced with permission [92]. Copyright 2009, American Chemical Society. (**e**) Schematic overview of biomimetic iron–fumarate metal–protein framework nanoparticles encapsulating diverse proteins. Reproduced with permission [102]. Copyright 2022, American Chemical Society. (**f**) Schematic representation of ZIF-8@BHb metal–protein framework composites formed by self-assembly. Reproduced with permission [82]. Copyright 2016, American Chemical Society.

Protein-coated metal nanoparticles represent one of the earliest and most widely used approaches [83]. Metallic nanoparticles such as gold, silver, platinum, and magnetic ferrites can be coated with a shell of serum albumin, ferritin, or other soluble proteins via adsorption or covalent conjugation, forming core–shell structures [41,84]. The protein shell has been shown to improve colloidal stability in aqueous and complex biological media and, in some experimental systems, to reduce nonspecific protein corona formation and reticuloendothelial clearance [85,86,87]; however, these effects are not universally observed across biological contexts [88]. The metal core, in turn, contributes photothermal, photoacoustic, X-ray, or magnetic resonance functionalities [89], so that the resulting core–shell constructs serve as commonly explored theranostic platforms in preclinical research settings with integrated imaging and therapeutic capabilities [90]. For example, Fang et al. encapsulated an oxygen-generating CaO_2_/catalase alginate core together with a ROS-producing ferric Schiff-base complex inside a human serum albumin shell to construct HSA–Fe1–O_2_ nanoparticles (Figure 3a,b). This protein-coated metal nanoplatform significantly enhanced intratumoral accumulation of the Fe complex, relieved hypoxia-induced resistance, and was associated with superior tumor suppression and limited systemic toxicity within the specific dosing regimens and tumor models evaluated compared with the free complex (Figure 3c) [91].

Protein-templated metal nanoclusters exploit coordinating residues within or on the surface of proteins to reduce and confine metal ions under mild conditions. A representative example is provided by Xie et al., who used bovine serum albumin (BSA) as a scaffold to synthesize highly stable gold nanoclusters with desirable optical properties (Figure 3d) [92]. In their investigations, Au(III) ions are first bound and sequestered by BSA in aqueous solution; increasing the pH to ~12 then activates the latent reducing ability of BSA, leading to stepwise in situ reduction in the bound ions and formation of Au nanoclusters that are stably embedded in the BSA matrix, where BSA serves simultaneously as reductant and stabilizer. Owing to pronounced quantum size effects, protein-templated metal nanoclusters often exhibit bright, tunable fluorescence and a high specific surface area with strong catalytic activity [93,94,95]. Nanoclusters templated by BSA, metallothionein, and related proteins have been applied in fluorescence imaging, ROS modulation, and antimicrobial therapy, and typically display good water solubility, acceptable biocompatibility, and in vivo stability in selected animal models owing to the protective, confining roles of the protein template [96,97,98].

Metal–protein frameworks (MPFs), a rapidly developing class of hybrid materials, represent another structurally complex and functionally rich family of metal–protein constructs. MPFs are typically formed through multidentate coordination between proteins and multivalent metal ions or metal clusters, combining the designable porosity of MOF-like architectures with the intrinsic biological functions of proteins [99,100]. Their internal pores can accommodate high loadings of diverse guest molecules, including small-molecule drugs, proteins, peptides, and nucleic acids, while the choice and spatial arrangement of metal nodes and protein units allow precise control over release profiles and stimuli responsiveness [101]. For instance, Mirzazadeh Dizaji et al. used a biomimetic mineralization strategy to encapsulate diverse proteins, including BSA, horseradish peroxidase, GFP, and Cas9/sgRNA ribonucleoproteins, into amorphous iron–fumarate nanoparticles, achieving high loading while preserving their catalytic and genome-editing activities after intracellular delivery (Figure 3e) [102]. Notably, these iron–fumarate MPFs protected Cas9 RNPs against acidic pH and long-term storage, underscoring the potential of MPFs as robust carriers for fragile protein therapeutics and gene-editing tools. As another example, Yin and co-workers used bovine hemoglobin (BHb) as the organic component and the zeolitic imidazolate framework ZIF-8 as the inorganic counterpart to construct BHb-embedded MOFs. The resulting ZIF-8@BHb composites, obtained by self-assembly, showed good stability and high catalytic activity and were further developed into a colorimetric platform for the visual detection of hydrogen peroxide (Figure 3f) [82]. Compared with traditional MOFs built from small organic ligands, MPFs based on native proteins usually exhibit improved biodegradability and biocompatibility in comparative preclinical studies, although systematic head-to-head evaluations and clinical validation remain limited [103].

### 2.4. Comparative Assessment and Design Principles

The above construction strategies reveal several general design principles governing metalloprotein-based nanomedicines. First, function is predominantly interface-driven: whether in native metalloproteins, engineered metal-binding sites, or hybrid nanostructures, the coordination environment at the metal–protein interface dictates metal speciation, redox activity, and stimulus responsiveness, thereby determining therapeutic and imaging performance [104,105]. Second, a fundamental stability–activity trade-off is consistently observed. Stronger protein confinement and higher-order assembly enhance colloidal stability, biocompatibility, and in vivo robustness but may simultaneously restrict metal accessibility or catalytic efficiency, necessitating careful balance through protein engineering or responsive coordination [106,107]. Third, controllability of metal speciation and coordination emerges as a central design axis. From evolutionarily optimized metal-binding pockets in native proteins to rationally redesigned coordination motifs and reversible metal-mediated self-assembly, precise regulation of metal valence states, coordination geometry, and release dynamics enables programmable activation and safer in vivo behavior [108,109]. These recurring principles suggest that successful metalloprotein-based nanomedicine design depends less on the nominal material class than on how effectively metal–protein interfaces are engineered to balance stability, activity, and biological compatibility [110,111].

How these general principles manifest in practice depends strongly on the chosen construction paradigm. From a comparative perspective, native metalloproteins, engineered metalloproteins, and metal–protein hybrid nanostructures share the common goal of integrating metal functionality with biological interfaces, yet differ markedly in stability, functional robustness, and translational attributes. Native metalloproteins benefit from evolutionarily optimized metal-binding sites and well-defined tertiary structures, which result in relatively favorable immunological profiles compared with many synthetic nanomaterials and predictable metabolism [112,113]. These features make them particularly attractive for applications requiring long circulation times and minimal immune perturbation. However, their functional scope is constrained by the intrinsic properties of the native metal cofactors, limiting tunability and the range of accessible therapeutic mechanisms [114]. Engineered metalloproteins offer increased design flexibility by allowing site-specific modulation of metal coordination environments, redox properties, and catalytic activity [115]. This enables precise tuning of function–structure relationships and facilitates modular optimization [116]. Nevertheless, such systems often face trade-offs between enhanced functionality and reduced structural robustness, and their immunogenicity and in vivo stability can be highly sensitive to sequence modifications and surface-exposed metal sites [117,118]. Metal–protein hybrid nanostructures provide the highest degree of functional integration, combining the rich physicochemical properties of inorganic nanomaterials with protein-mediated biocompatibility and targeting [69,119]. These systems have demonstrated strong therapeutic and imaging performance in multiple preclinical studies but also present greater challenges in terms of colloidal stability in biofluids, batch-to-batch reproducibility, scalable manufacturing, and regulatory evaluation, owing to their structural complexity and multicomponent nature [75]. Taken together, these three construction paradigms represent a continuum from biological fidelity to engineering freedom. Rational selection among them should be guided by application-specific priorities, balancing functional sophistication against stability, manufacturability, and translational feasibility. Importantly, these attributes are not interchangeable across application contexts; higher functional integration does not necessarily translate into superior in vivo performance when stability, immunological tolerance, or manufacturing constraints dominate design priorities (Table 1).

Overall, metal–protein hybrid nanostructures achieve spatial synergy between metal components and proteins at the structural level, while integrating imaging, therapy, and targeting functionalities at the functional level. With advances in designing protein architectures and engineering metal nanostructures, the application scope of these hybrid materials in nanomedicine is expected to expand further, and they are likely to become key building blocks for next-generation multimodal and intelligent therapeutic systems.

## 3. Biomedical Applications

Metalloprotein-based nanomedicines combine the multimodal imaging and therapeutic functions of metal centers with the finely tunable biointerfaces of protein scaffolds and therefore exhibit distinct advantages across multiple disease models. In the previous section, we outlined the design concepts of native metalloproteins, engineered metalloproteins, and metal–protein hybrid nanostructures from the perspective of structural types and construction mechanisms and established a basic classification framework based on metal-binding modes and protein scaffold features. Building on this, the present section summarizes representative advances from a function–disease viewpoint. It focuses on cancer therapy, imaging diagnostics, and multimodal theranostics; antimicrobial and anti-resistant therapy; and other disease interventions, with emphasis on the synergy between metal functional cores and protein-mediated fine regulation in specific applications.

### 3.1. Cancer Therapy

**Figure 4:**
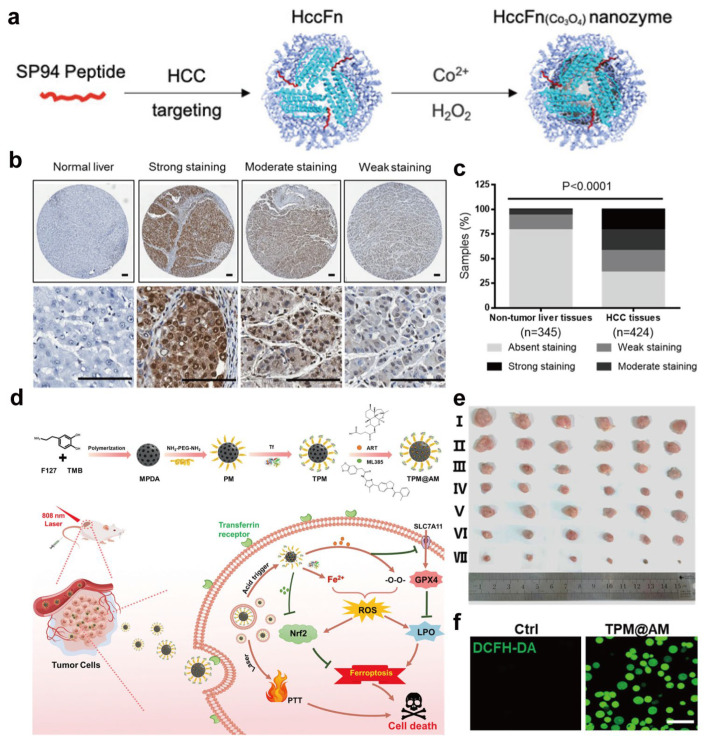
Metal–protein nanomedicines for targeted cancer diagnosis and therapy. (**a**) Schematic illustration of HCC-targeted ferritin-encapsulated Co_3_O_4_ nanozymes (HccFn(Co_3_O_4_)). (**b**) Immunohistochemical staining of HccFn(Co_3_O_4_) in tissue microarrays from HCC patients. Binding affinity was evaluated by staining index. Scale bar: 100 μm. (**c**) Quantitative comparison of HccFn(Co_3_O_4_) staining in HCC and nontumor liver tissues. Reproduced with permission [47]. Copyright 2019, American Chemical Society. (**d**) Schematic illustration of the transferrin-modified mesoporous polydopamine nanoplatform co-loading artesunate and ML385 (TPM@AM). (**e**) Representative photographs of excised tumors at day 12 (I: PBS, II: TPM, III: TPM@A, IV: TPM@AM, V: TPM/L, VI: TPM@A/L, VII: TPM@AM/L). Units: cm. (**f**) Confocal laser scanning microscopy (CLSM) images showing intracellular ROS generation in 4T1 cells. Scale bar: 50 μm. Reproduced with permission [126]. Copyright 2023, American Chemical Society.

Metal- and metalloprotein-based nanomedicines offer multi-dimensional synergies in cancer treatment. Encapsulating cisplatin, ruthenium complexes, or other metal-based drugs and clusters within protein nanocarriers has been shown to enhance tumor accumulation and mitigate off-target toxicity in selected preclinical models, although such benefits depend strongly on vascular permeability, protein stability, and tumor microenvironmental factors [43,120]. In addition to serving as simple carriers, metal–protein constructs can also function as catalytic nanoprobes for tumor diagnosis. For example, Jiang et al. biomineralized Co_3_O_4_ nanozymes inside hepatocellular carcinoma (HCC)-targeted ferritin nanocages (HccFn(Co_3_O_4_)) (Figure 4a), in which genetically displayed SP94 peptides on the protein shell confer high affinity for HCC cells and enable one-step immunohistochemical staining of clinical specimens with both diagnostic and prognostic value (Figure 4b,c) [47]. Passive targeting via the enhanced permeability and retention (EPR) effect has been reported to facilitate nanoparticle accumulation in tumors in certain animal models, although its relevance varies across tumor types and clinical settings [121,122,123]. Moreover, many protein scaffolds, such as transferrin and ferritin, can actively target tumor cells through high-affinity interactions with overexpressed receptors, thereby promoting receptor-mediated endocytosis and intracellular delivery in receptor-overexpressing cell models [54,124,125]. As an illustration of such receptor-targeted and microenvironment-responsive design, Chen et al. constructed a transferrin-modified mesoporous polydopamine nanoplatform co-loading the ferroptosis inducer artesunate and the Nrf2 inhibitor ML385 (TPM@AM), which selectively targets TfR-overexpressing tumor cells and releases the payloads in response to the acidic tumor microenvironment and NIR irradiation (Figure 4d) [126]. By increasing intracellular Fe^2+^ levels, boosting ROS and lipid peroxidation, and simultaneously suppressing Nrf2-mediated antioxidant defenses, TPM@AM markedly amplifies ferroptosis and achieves potent, spatiotemporally controlled tumor suppression in vivo with limited systemic toxicity observed under the tested conditions (Figure 4e,f).

In addition, the protein shell can modulate the local chemical microenvironment and metal coordination state, thereby tuning drug release kinetics and modes of action, while metal centers can participate in the Fenton and Fenton-like reactions to catalytically generate ROS and induce oxidative stress and apoptosis in tumor cells [96,127]. For example, Zhu et al. designed a ferritin-based nanoplatform (Ce6–PEG–HKN15-loaded ferritin) in which chlorin e6 (Ce6) acts as a photosensitizer [128]. Upon laser irradiation, Ce6 confined within the ferritin nanocages produces abundant ROS around the protein shell; the elevated ROS damages the iron-storage protein, triggers ferroptosis by releasing iron, and directly kills tumor cells. The liberated iron further reacts with excess intracellular H_2_O_2_ to generate O_2_, thereby relieving hypoxia, enhancing photodynamic therapy, and amplifying oxidative stress to achieve synergistic chemo-, catalytic, photothermal/photodynamic, and immunotherapy. Beyond such ferroptosis-oriented designs, other metal–protein nanoplatforms can elicit immunogenic cell death, promote tumor-associated antigen exposure, and facilitate immune cell infiltration, enabling combination regimens that integrate chemotherapy or catalytic therapy with immunotherapy and opening new avenues for multimodal cancer treatment [129,130,131].

However, the therapeutic mechanisms highlighted above are intrinsically associated with distinct risk profiles that warrant careful consideration alongside their benefits. Ferroptosis induction and ROS amplification, while effective in promoting tumor cell death, inherently increase the likelihood of off-target oxidative damage and systemic metal ion redistribution following protein degradation [132,133]. Moreover, tumor heterogeneity in redox balance, iron metabolism, and antioxidant capacity can lead to variable therapeutic responses, potentially limiting the robustness of Fenton- and ferroptosis-based strategies across different tumor models [134,135]. More broadly, each mechanism discussed in this section entails specific trade-offs: ROS amplification may cause collateral oxidative injury, hypoxia alleviation can alter vascular permeability, metal ion release may perturb systemic metal homeostasis, receptor-mediated targeting is susceptible to saturation and heterogeneity, and protein degradation may trigger unpredictable immune or metabolic consequences [136]. Recognizing these mechanism-linked risks is essential for realistic evaluation and rational optimization of metal–protein nanomedicines for cancer therapy.

### 3.2. Imaging Diagnostics and Multimodal Theranostics

**Figure 5:**
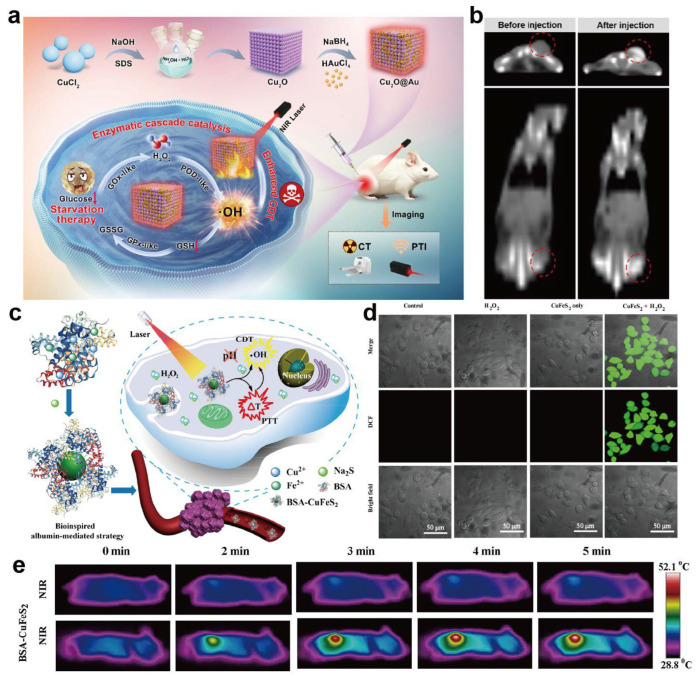
Metal–protein nanomedicines for imaging-guided theranostics. (**a**) Schematic illustration of the preparation of the Cu_2_O@Au nanozyme and its imaging-guided antitumor mechanism. (**b**) In vivo CT images before and after injection [148]. Copyright 2024, American Chemical Society. (**c**) Schematic illustration of the biomimetic synthesis of BSA–CuFeS_2_ nanoparticles and their pH-independent synergistic CDT/PTT. (**d**) Confocal fluorescence images of intracellular ROS generation in 4T1 cells after different treatments (DCFH-DA staining). Scale bar: 50 μm. (**e**) Representative infrared thermal images of 4T1 tumor-bearing mice during laser irradiation. Reproduced with permission [150]. Copyright 2019, American Chemical Society.

Metal–protein nanomedicines also show unique strengths in imaging diagnostics [137,138]. Metal ions serve as key contrast elements for many imaging modalities: Gd^3+^ and Mn^2+^ serve as T_1_ contrast agents in magnetic resonance imaging (MRI) [139,140], iron-based nanostructures act as T_2_ agents [141], and gold and other high-Z metals provide strong X-ray attenuation in computed tomography (CT) [142,143,144]. Metal nanoparticles further exhibit excellent photoacoustic signal conversion efficiencies for photoacoustic imaging [145,146,147]. For example, Li et al. designed a Cu_2_O@Au nanozyme that combines peroxidase (POD)-like catalytic activities with strong photothermal conversion and X-ray attenuation of nanoclusters (Figure 5a), enabling dual photothermal and CT imaging to guide cascade starvation/chemodynamic therapy in non-small-cell lung cancer models (Figure 5b) [148]. This work illustrates how metal-based nanozymes can simultaneously provide high-contrast imaging readouts and potent therapeutic effects within a single platform, enabling real-time imaging-guided and individualized treatment, potentially improving lesion localization and dose control when sufficient contrast-to-noise ratios and stable metal coordination can be maintained in vivo.

Integrating metal centers into protein scaffolds can greatly improve their aqueous solubility and in vivo stability. Moreover, protein-mediated targeting can facilitate preferential accumulation at diseased sites [149]. For instance, Chen and co-workers reported ultrasmall BSA–CuFeS_2_ nanoparticles as a multifunctional therapeutic platform prepared via a green, biomimetic albumin-mediated strategy (Figure 5c) [150]. These BSA–CuFeS_2_ NPs exhibit pH-independent Fenton-like catalytic activity for efficient ·OH generation and high photothermal conversion efficiency, thereby enhancing the combined efficacy of chemodynamic therapy (CDT) and photothermal therapy (PTT) (Figure 5d,e). The resulting hybrids display relaxivities comparable to clinical agents, excellent stability under physiological conditions, and negligible cytotoxicity, illustrating how protein engineering can be leveraged to tailor the magnetic and imaging properties of metal–protein nanomaterials. These advances in protein engineering lay the foundation for constructing multifunctional nanoplatforms capable of multimodal imaging and therapy integration.

When combined with fluorescent, photoacoustic, or nuclear imaging probes, these systems can be developed into multimodal imaging platforms that allow a single nanoconstruct to be used across MRI, CT, photoacoustic, and fluorescence channels in a complementary or synergistic manner [151,152]. By integrating imaging and therapeutic functions (e.g., MRI/PA/CT contrast, PTT, CDT, PDT, or chemotherapy) into a single construct, metal–protein nanomedicines potentially improve lesion localization and dose control when sufficient contrast-to-noise ratios and stable metal coordination can be maintained in vivo [10,149,153].

Nevertheless, the performance of metal–protein nanomedicines in imaging diagnostics and theranostics is also subject to mechanism-specific limitations [75]. Imaging contrast and quantitative accuracy critically depend on the in vivo stability of metal coordination and protein integrity [154]; partial metal dissociation, protein degradation, or dynamic protein corona formation can lead to signal attenuation, background noise, or misleading readouts over time [155,156]. In multimodal systems, differences in sensitivity, penetration depth, and temporal resolution among imaging modalities further complicate quantitative interpretation and cross-modality calibration [138,154]. Moreover, the introduction of therapeutic functions into imaging agents increases system complexity, potentially narrowing the safety margin and challenging dose optimization [157,158]. These considerations highlight the need for careful integration of imaging and therapeutic functions to ensure that enhanced multifunctionality does not compromise diagnostic reliability or translational robustness.

### 3.3. Antimicrobial and Anti-Resistant Therapy

**Figure 6:**
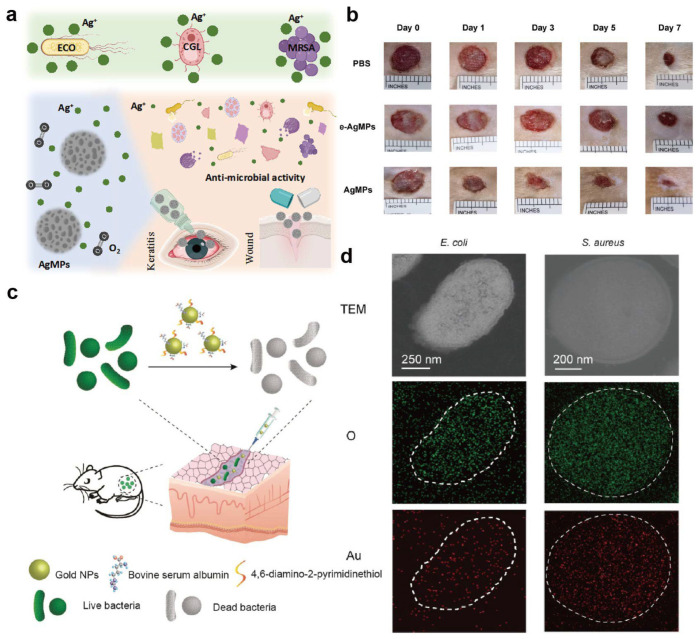
Metal–protein nanomedicines for antimicrobial and anti-resistant therapy. (**a**) Schematic illustration of protein-templated honeycomb-like silver microspheres (AgMPs) enabling sustained Ag^+^ release for broad-spectrum antimicrobial activity. (**b**) Photographs of CGL-infected wounds treated with PBS, pre-AgMPs or AgMPs at different time points (days 0–7). Reproduced with permission [161]. Copyright 2021, American Chemical Society. (**c**) Schematic illustration of the antibacterial mechanism of the BSA-stabilized gold nanocomposite (Au_DAPT_BSA) in the treatment of subcutaneous abscesses. (**d**) TEM images and elemental mapping (O, Au) of ultrathin sections of *E. coli* and *S. aureus* after treatment with Au_DAPT_BSA [162]. The colored overlays represent elemental mapping of O and Au, and the dashed circles indicate the boundaries of the Au_DAPT_BSA. Copyright 2019, American Chemical Society.

In the field of antimicrobial and anti-resistant therapy, metal–protein nanomedicines exert their effects through multiple mechanisms. Metal nanoparticles such as silver and copper can directly disrupt bacterial membranes, interfere with key metabolic enzymes, and induce excessive intracellular ROS accumulation [159]. Protein shells improve dispersion and biocompatibility and can be engineered to target and accumulate preferentially at infection sites, thereby enhancing local antibacterial efficacy in reported models while reducing systemic toxicity relative to unprotected metal formulations [160]. For example, Shi et al. prepared honeycomb-like silver microspheres (AgMPs) using bovine serum albumin as a template, which enable sustained Ag^+^ release and exhibit broad-spectrum activity against both bacteria and fungi, including *Candida* spp. (Figure 6a) [161]. In rat infection models, AgMPs effectively eliminated Candida-infected wounds while maintaining good viability of corneal epithelial cells at therapeutically relevant concentrations, highlighting the potential of protein-assisted silver nanostructures for treating fungal keratitis (Figure 6b).

Iron- or gold-containing metal–protein systems can catalyze the formation of highly reactive species such as hydroxyl radicals in bacteria-enriched regions, selectively killing drug-resistant strains and, to some extent, circumventing classical resistance mechanisms against conventional antibiotics. For example, Sun et al. synthesized a BSA-stabilized gold nanocomposite (Au_DAPT_BSA) that showed superior antibacterial activity against Gram-negative bacteria and improved efficacy against Gram-positive bacteria compared with Au_DAPT alone (Figure 6c) [162]. In mouse models of subcutaneous abscesses caused by clinically isolated multidrug-resistant *Escherichia coli* or *Staphylococcus aureus*, Au_DAPT_BSA markedly accelerated healing (Figure 6d).

Beyond the intrinsic antibacterial action of metal–protein hybrid materials, combination therapies have yielded more potent bactericidal effects and helped mitigate antibiotic resistance. For example, Liu et al. constructed a multifunctional antibacterial nanomedicine (BCPP) for targeted photodynamic/photothermal combination therapy (PDT/PTT) [163]. BCPP consists of protoporphyrin IX (PpIX) as the photosensitizer, CuS nanoparticles as the photothermal agent, phenylboronic acid (PBA) as a bacterial-targeting moiety, and BSA as the colloidal stabilizer. Owing to its strong singlet oxygen generation and high photothermal conversion efficiency, BCPP showed excellent targeting and killing efficacy against *S. aureus* and *E. coli*. Benefiting from these multi-mechanistic actions, metal–protein nanomedicines can not only improve antibacterial outcomes but also offer a means to delay or reduce the emergence of resistance. These systems therefore represent promising antimicrobial platforms; however, the same ROS-mediated and metal-ion-driven mechanisms that confer potent antimicrobial activity may also disrupt host cell redox balance or commensal microbiota, underscoring the need for spatial and dosage control [164,165].

Across cancer therapy, diagnostic imaging, and antimicrobial applications, several recurring design motifs emerge, including metal-mediated ROS generation, microenvironment-responsive activation, and protein-enabled multimodal functionality [166,167,168]. Rather than representing distinct strategies, these mechanisms often rely on shared physico-chemical principles, such as controlled metal confinement, protein degradation-triggered release, and environmental sensitivity [167,169]. Recognizing these commonalities helps clarify that differences among metalloprotein systems frequently arise from application context rather than fundamentally different design logic. Nevertheless, the same ROS-mediated and metal-ion-driven mechanisms that confer potent antimicrobial activity may also disrupt host cell redox balance or commensal microbiota, underscoring the need for spatial and dosage control [165,170].

### 3.4. Other Biomedical Applications

Beyond cancer and infection, metal- and metalloprotein-based nanomedicines also hold promise in neurodegenerative diseases, catalysis, and biosensing. In neurological disorders, dysregulated metal ion homeostasis and oxidative stress are recognized as key pathological drivers. Metal–protein nanoplatforms can modulate disease progression by chelating or slowly releasing metal ions and by regulating ROS levels [171,172]. For example, the accumulation of ROS, amyloid-β (Aβ), and neuroinflammation are hallmarks of Alzheimer’s disease (AD). Liu et al. designed ultrasmall copper nanoclusters embedded in human serum albumin (CuNCs@HSA) using an HSA-mediated strategy [173]. CuNCs@HSA exhibited multiple enzyme-like activities, significantly inhibited Aβ fibrillation, and alleviated Aβ-induced oxidative stress, thereby mitigating AD-related pathology in model systems.

The design of nanozymes that can compete with natural enzymes in catalytic efficiency while overcoming their limitations has attracted widespread attention. He et al. developed BSA–Pt nanoparticles in which platinum nanoparticles are supported on a BSA scaffold [174]. BSA–Pt displayed enhanced multi-enzyme-mimicking activities, including peroxidase-, oxidase-, and catalase-like functions. This work not only demonstrated the size-dependent nanozyme activity of Pt nanoparticles but also showcased the practical use of BSA–Pt for catalytic applications such as glucose detection in human serum.

As our understanding of the links between metal homeostasis and disease deepens, the application space of metal–protein nanomedicines in non-oncological indications is expected to expand further. However, the increasing diversity of therapeutic and diagnostic roles also brings new challenges in design, safety, and translational feasibility, which must be carefully addressed to realize their clinical potential.

## 4. Challenges and Future Perspectives

Despite the encouraging performance of metal- and metalloprotein-based nanomedicines in preclinical studies, their true clinical translation still faces multiple layers of challenges. These issues arise partly from incomplete basic understanding of nanoscale structures, metal–protein interfaces, and dynamic in vivo behavior, and partly from bottlenecks at the engineering and translational levels, including controllable fabrication, batch-to-batch consistency, long-term safety, and regulatory evaluation. Systematically identifying and addressing these challenges is essential for driving this class of nanomedicines from proof-of-concept toward tangible clinical benefit.

### 4.1. Metabolism and Degradation

**Figure 7:**
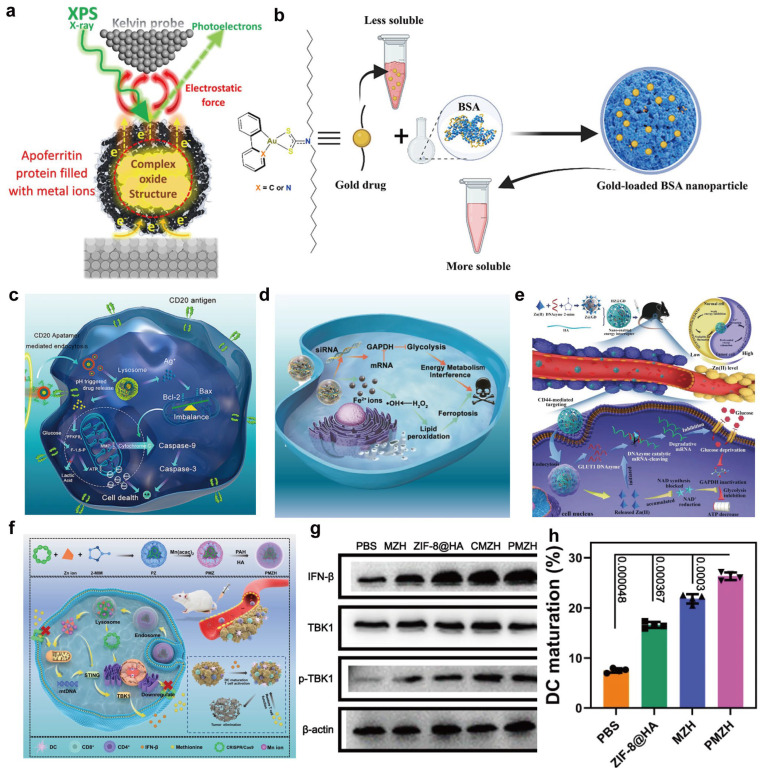
(**a**) Protein-mediated degradation of magnetic oxide nanoparticles. Interactions with apoferritin in the liver, spleen, and macrophages accelerate nanoparticle disassembly and metal ion release. Reproduced with permission [88]. Copyright 2024, American Chemical Society. (**b**) Degradation-regulated delivery of albumin-based metal drugs. Rationally engineered albumin nanocarriers improve solubility, intracellular accumulation, and metabolic stability of Au complexes via controlled scaffold degradation. Reproduced with permission [177]. Copyright 2023, American Chemical Society. (**c**) Schematic synthesis of PDA nanoparticles and (**d**) their interference with tumor energy metabolism. Reproduced with permission [180]. Copyright 2022 BioMed Central. (**e**) Dual gate-controlled “nano-enabled energy interrupter” that disassembles in the acidic, hyaluronidase-rich tumor microenvironment to co-release Zn^2+^ and a Zn^2+^-activated DNAzyme, synergistically inhibiting glycolysis and inducing systemic energy exhaustion. Reproduced with permission [181]. Copyright 2021, Wiley-VCH. (**f**) Schematic illustration of the PMZH nanoplatform for nutritional metal ion therapy, methionine metabolism regulation, and immune stimulation. (**g**) Immunoblot analysis of key STING pathway proteins in 4T1 cells after treatment. (**h**) Flow cytometric analysis of infiltrating dendritic cells following different treatments. Reproduced with permission [185]. Copyright 2023, Springer Nature.

The metabolism and degradation of metal- and metalloprotein-based nanomedicines in vivo are among the key determinants of both efficacy and safety. Metal centers typically exist as ions, nanoclusters, or oxides and may undergo valence changes, ligand exchange, or gradual dissolution in complex biological microenvironments, leading to slow release and redistribution of metal ions [175]. For example, magnetic oxide nanoparticles (MONPs) can interact with apoferritin in the spleen, liver, and inflammatory macrophages, which accelerates nanoparticle degradation and metal ion release and may alter the biological function of the protein partner (Figure 7a) [88]. Similarly, many nanomaterials undergo metabolism through a protein-corona-mediated cascade of transport, transformation, and bioavailability. Some nanomaterials containing essential trace elements may be converted into bioactive species and incorporated into normal metabolic pathways, but the long-term consequences of such transformations (e.g., metal ion accumulation or delayed toxicity) remain poorly understood and require careful investigation [176].

The degradation of the protein scaffold, including enzymatic digestion, denaturation, and partial unfolding, is equally important. Albumin-based nanocarriers, for instance, can be rationally designed with degradable features to improve the solubility, intracellular accumulation, and metabolic stability of metal drugs such as Au(III) complexes (Figure 7b) [177]. Differences in pH, ionic strength and enzyme expression between organs and within the tumor microenvironment (TME) can further lead to spatially heterogeneous metabolic pathways [178,179]. For example, metal–organic nanomaterials (MONs) can interfere with tumor metabolism by modulating glycolysis or oxidative phosphorylation, yet their degradation may be accelerated in acidic TME conditions (Figure 7c,d) [180]. In another study, Wu et al. report a dual gate-controlled ZIF-8/HA “nanoenabled energy interrupter” that disassembles in the acidic, hyaluronidase-rich TME to co-release Zn^2+^ and a Zn^2+^-activated DNAzyme. This coordinated Zn^2+^ overloading and GLUT1 mRNA cleavage synergistically blocks glycolysis, cuts off glucose supply, and drives systemic energy exhaustion in melanoma with minimal damage to normal tissues (Figure 7e) [181]. Moving forward, it will be important to systematically characterize the absorption, distribution, metabolism, and excretion of metal–protein nanomedicines in representative animal models, to distinguish the temporal–spatial distribution of different forms (metal ions, nanoclusters, protein fragments), and to quantitatively correlate metal release and protein structural changes with functional decay. Such knowledge will enable more predictive and tunable control over metabolic and degradation behaviors at the design stage.

### 4.2. Immunogenicity and Protein Recognition

As nanomedicines that incorporate proteins as major components, metal–protein systems are strongly influenced by immunogenicity and protein recognition in vivo, which directly impact both efficacy and safety. Protein source (autologous, xenogeneic, recombinant or de novo-designed), glycosylation and post-translational modifications, as well as the repertoire of exposed epitopes, all affect how the host immune system recognizes these constructs [182,183]. This recognition in turn shapes the formation of the plasma protein corona, uptake by the reticuloendothelial system (RES), and systemic circulation half-life.

Excessive immunogenicity can cause rapid clearance, neutralizing antibody production, or even hypersensitivity reactions, thereby compromising therapeutic benefit and raising safety concerns. Moreover, controlled immune activation can be harnessed to engage innate and adaptive immunity, offering additional leverage in anticancer and anti-infective therapies. Metal centers themselves may also modulate immune responses by altering cytokine expression, triggering the release of danger-associated molecular patterns, or changing antigen processing pathways, thereby indirectly affecting the type and intensity of immune responses [184]. Huang et al. reported an Mn/Zn-containing nanoplatform that synergistically elevated antitumor immunity by combining metal-ion-induced ROS amplification of tumor cells (Figure 7f), which was associated with enhanced dendritic-cell maturation and partial restoration of CD8^+^ T-cell function in the reported models (Figure 7g,h) [185]. This highlights the importance of finely tuning metal dosage and immune responses via optimized delivery systems to maximize therapeutic benefit while limiting systemic immune toxicity [186].

Consequently, it is necessary to systematically evaluate both short- and long-term immune effects of different protein scaffolds and metal loading strategies to distinguish undesirable immunogenicity from exploitable immunomodulatory outcomes [183,187]. At the molecular design stage, strategies such as selecting low-immunogenic protein frameworks, humanizing proteins, applying stealth surface modifications, and fine-tuning metal dosing should be employed to strike a rational balance between immune evasion and activation [188,189].

### 4.3. Controllable Preparation and Scale-Up Manufacturing

Controllable preparation and large-scale manufacturing represent significant engineering challenges on the path toward clinical translation of metal- and metalloprotein-based nanomedicines. Synthetic methods commonly used in the laboratory—such as chemical reduction, mineralization, self-assembly, and templated growth—are generally easy to handle at milligram or small-scale production. However, when scaled up to gram or larger quantities, they tend to reveal intrinsic weaknesses, including narrow process windows, limited yields, pronounced batch-to-batch variation, and broad distributions of particle size and metal loading [190]. Moreover, metal–protein systems are typically highly sensitive to temperature, pH, ionic strength, and shear, making it particularly difficult to maintain protein conformation and metal coordination environments consistently during scale-up [191].

Clinical translation demands manufacturing processes that are both reproducible and scalable and that meet strict quality control and traceability requirements. To this end, future process development should incorporate principles of transport and reaction engineering, process intensification, and continuous-flow operation to establish reaction systems and equipment configurations suitable for industrial production. In parallel, online monitoring and process analytical technologies (PAT) should be implemented to track critical quality attributes such as particle size distribution, metal loading, and protein integrity in real time, enabling feedback control and early fault detection [192]. Establishing standard operating procedures and quality evaluation systems in line with good manufacturing practice (GMP) guidelines will be key to achieving a smooth transition from laboratory formulation to engineered product [193,194]. It is worth noting that advances in chemical protein synthesis and semi-synthetic methodologies have already enabled the gram-scale production of artificial metalloproteins, providing a feasible route toward future industrial-scale manufacturing [195,196].

### 4.4. Clinical Translation

Beyond intrinsic material properties and manufacturing processes, clinical translation is further constrained by regulatory and evaluation-related factors. Metal–protein nanomedicines often comprise multiple components, hierarchical structures, and complex in vivo behaviors, which do not fit neatly into the existing regulatory frameworks developed for either small molecules or conventional biologics [75]. Current safety assessment protocols and in vitro surrogate models are largely designed for single metal ions or relatively simple nanomaterials and are not yet sufficient to fully capture the structural evolution and multi-pathway clearance of metal–protein hybrid systems in vivo, nor the associated potential risks [197,198]. A key unresolved challenge is how to systematically assess structural stability, metal–protein interface mechanisms, in vivo fate, long-term toxicity, and immune effects at the preclinical stage within reasonable cost and timelines [199,200].

In addition to challenges common to nanomedicines in general, metalloprotein-based systems face distinct barriers arising from the tight physicochemical coupling at the metal–protein interface [201]. Unlike polymeric or lipid-based nanocarriers, where drug loading and carrier integrity can often be optimized independently, metal–protein nanomedicines exhibit strong interdependence among protein folding, metal coordination geometry, and metal speciation [202]. Subtle variations in protein conformation, post-translational modification, or local microenvironment during production can propagate into changes in metal-binding affinity, redox state, and release kinetics, leading to amplified batch-to-batch variability that cannot be readily corrected by downstream formulation control [200,203,204].

Building on these interface-driven variabilities, additional challenges emerge from both manufacturing and regulatory perspectives. The dynamic and sometimes reversible nature of metal coordination—particularly for redox-active or labile metal centers—complicates the achievement of consistent metal loading [204,205], as metal ions or clusters often participate directly in structural stabilization or functional activation rather than behaving as passive cargo [206]. Consequently, metalloprotein-based nanomedicines do not fit neatly into existing regulatory frameworks for either biologics or inorganic nanomaterials [207]. The metal center may simultaneously function as a structural element, a catalytic site, and a pharmacologically active species, requiring regulatory evaluation to consider not only compositional consistency but also the stability and transformation of metal–protein interfaces under physiological conditions [208,209].

Moreover, differences in classification, submission pathways, and technical guidelines for nanomedicines across countries and regions further increase the complexity of global development and multi-center clinical trials [199,210]. Future efforts should focus on promoting the development of specific technical guidelines and evaluation standards tailored to the metal- and metalloprotein-based nanomedicines within the broader framework of regulatory science. Early and proactive dialogue with regulatory agencies and clinical stakeholders should be encouraged, and exploratory clinical trials and real-world evidence studies should be leveraged to gradually build robust safety and efficacy data, thereby paving the way for standardized approval pathways.

Taken together, the translational prospects of metal- and metalloprotein-based nanomedicines are governed by a complex interplay among metal chemistry, protein scaffold properties, mechanism of action, and regulatory constraints. To facilitate cross-comparison and to provide a consolidated view of how these factors jointly shape biomedical performance and translational bottlenecks, Table 2 summarizes representative metal–protein systems by linking metal type, protein scaffold, dominant mechanism, biomedical application, and key translational challenges discussed throughout this review.

### 4.5. Rational Design and AI-Enabled Engineering

Several limitations identified throughout this review—including the difficulty of predicting in vivo metal release, the immunogenicity of engineered protein surfaces, and the long-term biodistribution and fate of metal–protein hybrids—remain poorly addressed by empirical approaches alone [211,212,213]. These challenges repeatedly emerge across native metalloproteins, engineered metal-binding proteins, and metal–protein hybrid nanostructures, reflecting the complex, non-linear coupling between protein structure, metal coordination, and biological context [211]. In this regard, recent advances in AI-assisted protein design and data-driven modeling offer promising tools to complement traditional experimental strategies by integrating structural, biochemical, and in vivo datasets, although they currently remain limited in capturing non-equilibrium, multiscale, and patient-specific dynamics [155,214].

Beyond conceptual appeal, AI-enabled approaches can be explicitly linked to several unresolved problems highlighted in earlier sections of this review. One prominent example is the prediction of in vivo metal release, which remains challenging due to the context-dependent stability of metal–protein coordination under physiological forces, redox gradients, and competitive ligand environments [215]. Data-driven models trained on protein sequence–structure–metal affinity relationships could help identify coordination motifs, thereby narrowing the gap between in vitro metal loading efficiency and in vivo release behavior observed in therapeutic and diagnostic applications [216,217]. Immunogenicity represents another major translational uncertainty, particularly for engineered metalloproteins and metal–protein hybrid nanostructures with non-native surfaces [218]. Machine-learning approaches that integrate sequence features, surface epitope exposure, protein corona composition, and prior immunogenicity datasets may enable early-stage screening of variants with reduced immune recognition [219,220]. Such predictive tools could complement low-throughput and costly empirical immunoassays and are especially relevant for repeat-dosing regimens, where subtle sequence or structural modifications can disproportionately affect immune memory, clearance, and safety profiles [221,222]. Long-term biodistribution and clearance pose additional challenges, especially for ferritin-, albumin-, and protein–inorganic hybrid systems whose degradation pathways and metal fate remain incompletely understood [61,223,224]. AI-assisted pharmacokinetic and biodistribution modeling, informed by protein size, surface charge, metal content, coordination stability, and degradation kinetics, may assist in forecasting organ accumulation patterns and metal redistribution over extended timeframes [225]. Such models could help guide rational design toward safer and more predictable in vivo behavior, addressing concerns raised earlier regarding systemic metal homeostasis and off-target accumulation.

Importantly, the growing availability of high-resolution experimental techniques provides the foundation upon which such AI models can be meaningfully trained and validated. Cryogenic electron microscopy (cryo-EM), for example, has enabled near-atomic visualization of metal–protein interfaces by exploiting the strong scattering signals of transition metals, allowing direct observation of coordination environments and structural evolution [226]. Protein footprinting and related spectroscopic methods further complement cryo-EM by probing metal–protein interactions and conformational dynamics in complex or heterogeneous systems that are otherwise difficult to resolve [227,228]. These approaches are essential for establishing quantitative structure–function relationships that underpin reliable data-driven modeling.

In parallel, AI-assisted tools for protein structure prediction and sequence design have begun to demonstrate practical utility in metalloprotein engineering [229,230]. Approaches such as SPDesign enable backbone-based protein sequence optimization using structural–sequence profiles and ultrafast shape recognition, accelerating the development of functional proteins, including artificial metalloproteins [231]. Machine-learning and deep-learning models have achieved high accuracy in predicting metal-binding sites in proteins and are being extended to model nanoparticle–protein interactions [110,232,233]. Strategies such as MASCoT further illustrate how geometric constraints can be exploited to design artificial metal coordination environments with defined ion specificity [65]. At a higher level, interpretable ML models trained on large datasets of inorganic nanoparticle studies have begun to identify key parameters governing in vivo behavior and toxicity, offering transferable insights for metal–protein nanomedicine optimization [234,235].

**Figure 8:**
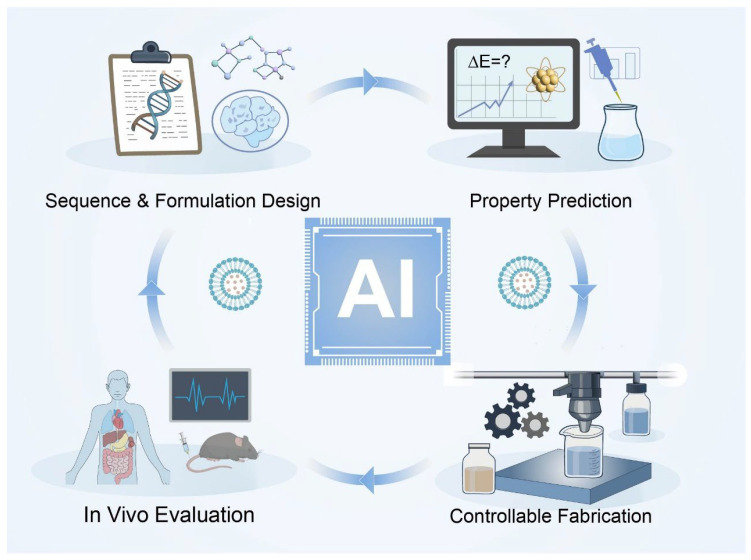
Overview of current challenges and future perspectives in metal–protein nanomedicine.

When integrated with automated synthesis platforms, online process analytics, and high-throughput in vitro and in vivo screening [236,237], these AI-enabled tools may enable closed-loop R & D workflows spanning sequence and formulation design, property prediction, controllable fabrication, and biological evaluation [225,238]. Such frameworks have the potential to shift metalloprotein-based nanomedicine development from empirical trial-and-error toward data-driven, model-guided engineering [239]. Nevertheless, AI-assisted approaches should be viewed primarily as hypothesis-generating and decision-support tools rather than definitive solutions, as their predictive power remains constrained by data quality, dataset diversity, and the intrinsic complexity of in vivo biological systems [240,241]. Continued integration of experimental insight, mechanistic understanding, and data-driven modeling will be essential for translating these advances into clinically robust metal- and metalloprotein-based nanomedicines (Figure 8).

## 5. Conclusions and Outlook

Metal- and metalloprotein-based nanomedicines integrate the multifunctionality of metal centers with the engineerability of protein scaffolds, enabling the construction of advanced nanoplatforms that combine targeting, controlled release, and multimodal functions. These systems have demonstrated potential advantages in pharmacokinetics, targeted accumulation, and functional synergy in preclinical studies, and promising efficacy in a range of pre-clinical studies [75]. In particular, metal nanocluster–protein conjugates, owing to their atomically defined structures and cooperative effects between the metal core and protein shell, exhibit strong and versatile performance in bioimaging, biosensing, and catalysis [155]. In this review, we have provided a systematic overview of current metal–protein nanomedicines from three perspectives: material types and construction mechanisms, biomedical applications, and key challenges, together with future opportunities.

Despite these advances, this field faces three major intertwined challenges: (i) At the level of fundamental mechanisms, interactions at metal–protein interfaces are not yet fully understood. Specifically, binding modes, structural dynamics, and structure–function relationships require deeper elucidation using integrated experimental and computational approaches [155]. (ii) At the level of process engineering, scalable and reproducible manufacturing remains difficult, and more robust and controllable synthetic routes are needed to meet quality requirements for clinical development [242]. (iii) At the translational level, long-term safety, immunogenicity, and complex in vivo biological effects constitute major barriers that must be addressed through toxicity and immunology studies [243]. In addition, the stability, immunogenicity, and manufacturability of protein-based nanoparticles themselves remain important constraints that need careful consideration in material design [75,244].

Future progress will require a paradigm shift from empirical trial-and-error approaches toward rational, data-driven design. Artificial intelligence-assisted protein design, computational chemistry, and multiscale simulations—coupled with high-throughput and automated experimentation—are emerging as powerful tools to drive this transformation [245,246]. Integrating these capabilities with advances in protein engineering, metal nanoscience, and process development is expected to enable closed-loop workflows that link sequence and formulation design, property prediction, controllable fabrication, and in vivo evaluation. We anticipate that, as fundamental understanding deepens and enabling technologies mature, metal- and metalloprotein-based nanomedicines—combining inorganic functionality with biological intelligence—will move closer to clinical translation and have the potential to become important components of future precision medicine strategies, pending further validation.

## Figures and Tables

**Table 1:** Comparative evaluation of metalloprotein-based nanomedicine construction strategies.

Strategy	Stability in Biofluids	Functional Tunability	Immunological Behavior	Manufacturability	Translational Feasibility
Native metalloproteins	High	Limited	Generally favorable	High	High
Engineered metalloproteins	Moderate–high	High	Variable, design-sensitive	Moderate	Moderate
Metal–protein hybrids	Variable	Very high	More complex	Challenging	limited

**Table 2:** Representative metalloprotein-based nanomedicines categorized by metal type, protein scaffold, interface-driven mechanism, biomedical application, and key translational bottlenecks.

Metal Type	Protein Scaffold	Interface Mechanism	Key Advantages	Major Limitations	Translational Bottleneck
Fe	Ferritin	Biomineralization	High biocompatibility, targeting	Limited loading flexibility	Scale-up, batch variability
Fe	Transferrin	Native coordination	Receptor-mediated uptake	Receptor saturation	Patient heterogeneity
Au	Albumin	Protein templating	Stability, fluorescence	Limited metal exposure	Long-term fate
Cu/Fe	Engineered proteins	Designed coordination	Tunable activity	Structural complexity	Manufacturability

## Data Availability

No new data were created or analyzed in this study.
